# Prevalence and Factors Associated with Problematic Internet Use among Ethiopian Undergraduate University Students in 2019

**DOI:** 10.1155/2021/6041607

**Published:** 2021-12-09

**Authors:** Nebiyu Mengistu, Desalegn Tarekegn, Yesuneh Bayisa, Solomon Yimer, Derebe Madoro, Dawit Getachew Assefa, Eden Dagnachew Zeleke, Wondwosen Molla, Aregahegn Wudneh, Seid Shumye, Bereket Duko

**Affiliations:** ^1^Department of Psychiatry, Dilla University, Dilla, P.O. Box (DU): 419, Ethiopia; ^2^Department of Midwifery, Dilla University, Dilla, P.O. Box (DU): 419, Ethiopia; ^3^School Medicine, Dilla University, Dilla, P.O. Box (DU): 419, Ethiopia; ^4^Department of Nursing, Dilla University, Dilla, P.O. Box (DU): 419, Ethiopia; ^5^Department of Nursing, Bulle Hora University, Bulle Hora, Ethiopia; ^6^Curtin School of Population Health, Curtin University, Perth, WA, Australia

## Abstract

**Background:**

Problematic Internet use is characterized by excessive or poorly controlled preoccupations, urges, or behavior regarding computer use and Internet access which lead to impairment or distress. It has been found that the occurrence rate of problematic Internet use among university students ranges from 0.8% to 47.7%. Despite this, there are multiple challenges that relate to problematic Internet use, which remain underrecognized and largely ignored by stakeholders and are not well known, especially in low-income countries, including Ethiopia. Therefore, this study was conducted aiming to assess the prevalence of problematic Internet use and its associated factors among undergraduate students.

**Methods:**

Cross-sectional study was employed from May 1st to June 1st, 2019. A multistage sampling technique was used to get a total of 846 undergraduate students. Data were collected by using self-administered structured questionnaires of Young's Internet Addiction Test. The collected data were coded and entered into EpiData 3.1 and analyzed by using SPSS version 22; bivariate and multivariate logistic regression analysis was conducted to identify factors associated with problematic Internet use, and statistical significance was considered at *P* value <0.05.

**Results:**

For a total of 846 study participants, the response rate was 761 (90%) and the prevalence of problematic Internet use was 19.4%. Multiple logistic regression model revealed that being male [AOR = 1.69, 95% CI: 1.80, 6.41], depression [AOR = 3.61, 95% CI: 2.40, 5.43], and khat or caffeinated drinks [AOR = 1.86, 95% CI: 1.21, 2.87] were significantly associated with problematic Internet use.

**Conclusion:**

This study revealed that there was high prevalence of problematic Internet use among Dilla University students and there were various factors associated with increased prevalence of problematic Internet use. Therefore, students need to be educated about the safe, valuable, and healthy practices of Internet use. Furthermore, it is better to counsel on substance use and its consequences to overcome the anticipated increase in problematic Internet use.

## 1. Introduction

Internet is growing worldwide for information and is a user-friendly communication medium that is a cost-effective and useful tool in education [1]. Internet technology has changed our daily lives dramatically. Adolescents and young adults, in particular, may be attracted to and preoccupied with various online activities [2, 3].

Problematic Internet use is defined as the “inability to stop Internet overuse, a tendency to perceive offline time as meaningless, excessive irritation, and aggression during deprivation” and can be also described as Internet dependence, pathological Internet use, or compulsive Internet use [4, 5]. It is a new and attractive subject considered as a behavior-based addiction in recent years [6]; and it is becoming a serious problem across the world, especially for adolescents. Scholars have also warned that problematic Internet use could cause a substantial loss of productivity in schools and companies. On the basis of the existing literature, it was hypothesized that problematic Internet use would have a negative impact on the academic performance of university students [7, 8].

It has been found that the occurrence rate of problematic Internet use among university students ranges from 0.8% to 47.7% [9–17]. Most study findings have shown that excessive use of the Internet adversely affects one's physical health, family life, and academic performance. Academic problems caused by problematic Internet use include a decline in study habits, a significant drop in grades, missing classes, an increased risk of being placed on academic probation, and poor integration into extracurricular activities [17–21].

University students, more than any other segment of society, are particularly vulnerable to acquiring problematic Internet use. This can be attributed to a variety of factors, including time availability, ease of use, psychological and developmental characteristics of young adulthood, limited or no parental supervision, and an expectation of covert Internet/computer use, if not from assignments and projects, as some courses are Internet-dependent; the Internet offers a way to escape from exam anxiety [22]. Heavy Internet use has many associations with depression, poor sleep quality, mood changes, and poor health outcomes such as obesity and low self-esteem [23].

Studies in different countries have generated widely different estimates. There was very low prevalence of problematic Internet use among college students, for example, in Italy (0.8%) (8), whereas the prevalence rate of problematic Internet use as high as 18% has been reported in the UK [11].

A survey conducted in Asian countries like Japan, China, Pakistan, and India revealed rates of 21.6%, 26.7%, 16.7%, and 18.88%, respectively. The highest problematic Internet use risk profile is that of a male, under the age of 21, with low self-esteem who lives away from home, making him more vulnerable to problems and also to depression and anxiety [13–15, 24].

Another cross-sectional survey conducted on Internet addiction among medical students at Sohag University in Egypt revealed that the prevalence of problematic Internet use was 47.7%. Being male, mobile phones access to Internet, easy Internet access at home, using the Internet for browsing social media and e-mail, and bad relationships with family were the most important predictors of problematic Internet use [25].

Even though problematic Internet use is becoming a serious problem across the world, especially for adolescents [26], scholars have also warned that problematic Internet use could cause substantial loss of productivity in schools and companies and significant health problems because of increased social media use among the early adult population and growing Internet access [5, 27], but, to of the investigator's knowledge, the prevalence of problematic Internet use and its associated factors among undergraduate students are not well studied in Ethiopia as well as in the current study area.

Besides that, the current study addressed key correlated factors that could be managed by stakeholders to provide information for students, the need to be educated about the safe, valuable, and healthy practices of Internet use, and the control of psychological problems among students.

Therefore, this study will aim to assess the prevalence of problematic Internet use and its associated factors among undergraduate university students.

## 2. Methods and Materials

### 2.1. Study Design, Period, Setting, and Population

An institutional-based cross-sectional study was conducted at Dilla University located 360 km south of the capital city of Ethiopia from May 1, 2019, to June 1, 2019. One of the newer universities that was founded in 1996 was named as Teachers and Health Science College in Ethiopia. However, since 2007, it has been providing a higher level of education in many disciplines, which has been clustered into three campuses and six colleges. Currently, it has 47 undergraduate and 17 postgraduate departments at Bachelor of art/Bachelor of science and Master of art/Master of science levels with regular extension and summer courses, and it has about 30,108 students: 18,452 males and 11,656 females. The number of regular undergraduate students in 2018/2019 was 19,350: 11,506 males and 7844 females. The study population consisted of undergraduate students from Dilla University who were in their first to the sixth years of study.

### 2.2. Inclusion and Exclusion Criteria

The subjects included in this study were all randomly selected undergraduate students of Dilla University who were in their first to the sixth years of study whose age was greater than 18 years and they were available during data collection, whereas those who were critically ill were excluded from the study.

### 2.3. Sample Size Determinations, Sampling Techniques, and Procedures

The sample size of this study was calculated using single population proportion formula and the total sample was 384. By considering a 10% nonresponse rate and design effects of 2, the final sample size became 846.

In Dilla University, there are six colleges, namely, college of technology and engineering, college of business and economics, college of medicine and health sciences, college of social science and humanities, college of agriculture and natural resources, and college of natural and computational science, and two institutes (one is indigenous studies and the other is education and behavioral science), as well as separate schools of law. We used a multistage cluster sampling procedure to select a sample of undergraduate students. Initially, two colleges (college of medicine and health sciences and college of business and economics) and one school (school of law) were selected by using simple random sampling technique (lottery method). In the second stage, the selected college and school were stratified based on the departments. There are thirteen departments in the selected colleges and schools. All these departments with their level of academic years (batches) were included in this study and the design effect was used. The final sample size was allocated proportionally for each department based on the number of their students with their level academic years (batches). Finally, a simple random sampling technique was used to select participants by using their ID number as a sampling frame.

### 2.4. Data Collection Tools and Procedures

Self-administered, well-structured, and organized English version questionnaire was disseminated to students, and data were collected from each student at classroom and this process lasted between 30 and 40 min. The questionnaires comprised nine parts; the first part consisted of sociodemographic details; a structured questionnaire was used to assess sociodemographic characteristics. The second part includes Internet access and experience related factors which had three items; the third part consisted of data on the outcome of interest, that is, the problematic Internet use, which was collected by using Internet Addiction Test (IAT). This scale has been widely used for screening and measuring the level of Internet addiction worldwide, and IAT showed that it is more reliable in university students. Generally, Cronbach in the present study was 0.89. Each item in the IAT 20 is rated on a scale from rarely to always. Using a five-point Likert scale, the responses were assigned a numeric value or score where a score of one indicated “rarely” and a score of five indicated “always.” These items include questions about compulsive behavior related to the use of the Internet, the presence of problems with academic performance, bad home environments, relationships and problems with family or friends, and suffering from emotional problems. After answering all the questions, the scores of each response are added together to obtain a final score ranging between 20 and 100. The higher the score, the greater the level of addiction. There is no gold standard for distinguishing between PIU and nonusers. According to the IAT manual, users are given four labels based on the total score: normal user (score of 20), mild users (score between 20 and 49), moderate users (score between 50 and 69), and severe or excessive users (score of 80) [28]. Since moderate users are often unable to control their Internet use; we considered both moderate and excessive use of the Internet (IAT total score of 50) as problematic Internet use and, based on the PIU test manual, we considered that those who scored between 20 and 49 were normal Internet users. This opinion of problematic Internet use classification is fairly supported by the existing literature [25, 29, 30].

The fourth part consisted of Hamilton Anxiety Rating Scale (HARS) which was used to measure the severity of anxiety symptoms and is still widely used today in both clinical and research settings. For HARS total score, the intraclass correlation measure of interrater reliability was 0.92 and the value of Cronbach's alpha was 0.86. The scale consists of 14 items. Each item is scored on a scale of 0 (not present) to 4 (severe), with a total score range of 0–56, where 17 indicates mild level, 18–24 mild to moderate level, and 25–30 moderate to severe level. Participants with a score of 18–27 were considered normal, while those with a score of 25–30 (moderate to severe) were considered to have probable anxiety [31].

The fifth part consisted of the Hamilton Depression Rating Scale (HDRS) which is the most widely used clinician-administered depression assessment tool. It is a 17-item scale questionnaire pertaining to symptoms of depression experienced over the past week. For nonclinical participants, test-retest coefficient was 0.82 for HDI-17 and the internal consistency reliability of all forms of the HDRS was high and ranged from 0.91 to 0.94. Individuals with a score of 0–7 are considered to be in the normal range, whereas those with a score of 20 or more are considered to be probably depressed [32].

The sixth part consisted of Rosenberg self-esteem (RSE) scale which was used to assess self-esteem. It was measured on a 10-item scale questionnaire with a 4-point Likert scale format ranging from 1 to 4 (strongly agree, agree, disagree, and strongly disagree). Its test-retest reliability over a period of 2 weeks reveals correlations of 0.85 and 0.88, indicating excellent stability. Accordingly, low self-esteem responses are operationalized when individuals respond with “disagree” or “strongly disagree” for items 1, 3, 4, 7, and 10 and “strongly agree” or “agree” for items 2, 5, 6, 8, and 9. Two or three out of three correct responses to items 3, 7, and 9 are scored as one item. One or two out of two correct responses for items 4 and 5 are considered as a single item; items 1, 8, and 10 are scored as individual items; and combined correct responses (one or two out of two) to items 2 and 6 are considered to be a single item [33].

The seventh part consisted of peer pressure which was measured by the PPQ-R, which is a 29-item self-report scale that assesses peer influence in everyday life situations. Cronbach's alpha coefficient was *r* = 0.91. For test-retest reliability, the correlation coefficient between the two applications in total scores was *r* = 0.85. It is a 5-point Likert scale with scores from 1 (strongly disagree) to 5 (strongly agree). Individuals with the highest score (above the study's mean of 21.4 points) were under higher peer pressure [34]. The eighth part consisted of social support which was measured using the Oslo 3-item social support (OSS-3) scale. Individuals with a score of “3–8” had poor social support, those with a score of “9–11” had intermediate support, and those with a score of “12–14” had strong support [35]; and the last part consisted of the psychoactive substance use-associated factors; a self-report questionnaire was used to assess the current use of psychoactive substances (khat or caffeinated drinks, tobacco, and alcohol) [36].

### 2.5. Data Quality Control Issues

Training was given to the data collectors and supervisors on the data collection tools and sampling techniques. Supervision was held regularly during the data collection period, by the researcher, coinvestigators, and supervisors, to check daily for completeness and consistency. In addition, a pretest of the study was carried out on 5% (43) of the total undergraduate students from outside of the study area (Hawassa University, which is closer to the study area).

### 2.6. Data Processing and Analysis

Following the accomplishment of data collection activities, the questionnaire was entered into EpiData version 3.1 (where QES, REC, and Check files were created) to ensure a double data entry system and then it was exported to SPSS version 22 to accomplish further data exploration procedures, along with the required statistical data analysis methods. Descriptive statistics (mean, median, frequency, and percentage) were used to summarize data and the results were reported using frequencies, percentages, charts, and tables. Bivariate and multivariate logistic regression analysis was conducted to identify factors associated with problematic Internet use and statistical significance was considered at *P* value <0.05.

## 3. Results

### 3.1. Sociodemographic Characteristics of Respondents

There were a total of 846 study participants, giving a response rate of 761 (90%). However, the remaining 10% (*n* = 85) of participants were excluded due to incomplete responses. The mean age of the respondents was 20.74 years with SD of 1.79; the minimum and maximum ages of the participants were 18 years and 30 years, respectively. The proportion of male to female participants was 59.8% to 40.2%. More than half of the students (65.7%) were Orthodox by religion and most of the respondents were single (66.1%) regarding marital status (Table 1).

### 3.2. Internet Access and Experience of the Participants

Many of the study participants had a practice of using the Internet for more than twelve months, 453 (59.5%). About 475 (62.4%) and 438 (57.5%) were using the Internet less than five hours per day and used their mobile data for Internet access, respectively (Table 2).

### 3.3. Psychosocial and Behavioral Related Characteristics of the Participants

In terms of psychosocial and behavioral characteristics, 178 (23.4%) of the students were depressed, 213 (28.0%) of the participants exhibited anxiety disorder symptoms, and 307 (40.3%) of the students had poor social support; and 419 (55.1%) of those students reported low self-esteem, whereas 421 (56.4%) had lower peer pressure (Table 3).

### 3.4. Psychoactive Substance Use Related Factors of Respondents

The current use of substances among 761 study participants was 490 (64.4%). Among those substance users, the majority (202 (26.5%)) used alcohol, followed by 158 (20.8%) using khat and caffeinated drinks and 130 (17.1%) with current use of tobacco.

### 3.5. Prevalence of Problematic Internet Use

From the total of 761 Dilla University undergraduate students, 80.6% were normal Internet users (scores ≤ 49); 16.8% were classified as moderate addicts (scores 50–79); and those who were severe addicts represented 2.6% of participants with a score of at least 80. Students classified as moderate and severe Internet addiction were considered problematic Internet use users (scores from 50 to 100) and represented 148 (19.4%) with 95% CI of 16.6, 22.5 and those with lower scores (scores ≤49) were considered as normal users of Internet.

### 3.6. Factors Associated with Problematic Internet Use among Undergraduate Students

In the bivariate analysis from sociodemographic variables (age and sex), psychosocial and behavioral related factors (depression, anxiety, and social support) and current use of khat or caffeinated drinks and self-esteem fulfilled the minimum requirement (*P* < 0.25) and were taken for further multivariate analysis. On the other hand, peer influence and current use of tobacco and alcohol did not fulfill the minimum requirement and were excluded from further analysis.

After multivariate analysis of problematic Internet use in relation to all independent variables, male sex, probable depression, and current use of khat or caffeinated drinks were found to be statistically significant.

The prevalence of problematic Internet use was found to be 1.69 times higher in male undergraduate students than in females (AOR = 1.69, 95% CI: 1.12, 2.56).

The odds of developing problematic Internet use were more than three times higher among depressed respondents compared to nondepressed ones (76.6% versus 23.4%) [AOR = 3.61, 95% CI: 2.40, 5.43].

Regarding psychoactive substance use, those who currently use khat or caffeinated drinks were 1.86 times more likely to develop problematic Internet use as compared to those who did not use these substances (AOR = 1.86, 95% CI: 1.21, 2.87) (Table 4).

## 4. Discussion

The current study aims to assess the prevalence and associated factors of problematic Internet use among undergraduate university students in Ethiopia. In this study, 761 undergraduate students were surveyed to ascertain the prevalence of problematic Internet use and its associated factors and this study revealed that the prevalence of problematic Internet use was 19.4% with 95% CI of 16.6, 22.5. Being male, probable depression, and currently using khat or caffeinated drinks were significantly associated with PIU. The present study is in line with studies conducted in India [14], Pakistan [15], Japan [24], and the United Kingdom [37] among university undergraduate students, which had rates of 18.8%, 16.7%, 21.6%, and 18%, respectively. This consistency may be due to sharing of the same study population with the same age group used, as well as the use of the same assessment method (the IAT) to classify problematic Internet use.

The prevalence of problematic Internet use in the present study was higher than those in the studies done in various countries such as Italy (0.8%) [38], Iran (7.3%) [37], Spain (6.08%) [9], and Lebanon (16.8%) [39]. The discrepancy might be due to sociocultural differences such as time utilization in the study population, the cutoff point of YIAT-20, instrument differences, mental health policies, and differences in study participants; for example, in our study, the participants were from two colleges and one school. Moreover, the age of the study participants varied from one country to another (in Spain, the participants were over 21 years old). Another possible explanation may be time differences between past and present studies, due to the accessibility of social media sites like Viber, Twitter, TikTok, and Instagram, which are the main reasons people use the Internet [40]. However, the finding of the current study was lower than those of studies done in Egypt (47.7%) [25], Saudi Arabia (31.7%) [16], China (26.7%) [13], Nepal (42%) [17], and Greece (32.2%) [41]. This difference could be attributable to the longitudinal study design used in the United States. Other distinctions include participant socioeconomic inequalities, such as easy Internet access at home and on mobile phones, and the study's sample size (*n* = 2200) in Greece.

In the present study, the prevalence of problematic Internet use was found to be 1.69 times higher in males than in females [AOR = 1.69, 95% CI: 1.12, 2.56]. This result is supported by studies carried out in Egypt [25] and Iran [37]. The possible justification for this strong association could be explained by the fact that males are more likely than females to have a high level of web familiarity and also the fact that men receive less parental supervision and they use the Internet for entertainment purposes more than women [42, 43]. On the other hand, the present findings disagree with studies done in France [44]. The discrepancy might be due to sociocultural differences and variation in the age of study participants. In addition, the variation in sample size (higher frequency of males, which is (73.5%)) is also considered.

Likewise, students who had depression were more than three times as likely to develop problematic Internet use as compared to their counterparts (76.6% versus 23.4%) [AOR = 3.61, 95% CI: 2.40, 5.43]. Study findings in these areas showed that students who had depression were more related to problematic Internet use than students who did not have depression. This finding is in agreement with studies done in Iran [37], India [45], and Turkey [24]. The possible reason for this may be that individuals suffering from depression can have the predisposition to have problematic Internet use. A depressed mood can impair coping with stress and may make subjects escape the negative experience by entering the Internet. Therefore, students who have depressive symptoms usually seek the Internet for socializing with distant friends and short-term relief, which makes them addicted to the Internet [20, 46, 47].

Among substance users, current use of khat or caffeinated drinks was significantly associated with problematic Internet use, which is 1.86 times more likely to lead to problematic Internet use compared to no use of these substances [AOR = 1.86, 95% CI: 1.21, 2.87]. This finding agrees with studies conducted in Iran [30] and on Greek university students [48]. This may be explained by the fact that the biological effect of the substances on the brain is that khat or caffeinated drinks are central nervous system stimulants that have the ability to enhance alertness and concentration, boost mood and motivation to work, and reduce the craving or compulsive effects of the substances, which are also associated with symptoms of problematic Internet use. Therefore, many people might be easily motivated or urged to use the Internet [49].

### 4.1. Limitation

This study does not show any cause-effect relationship because of the cross-sectional nature of the study design, and there are also chances for social desirability bias, wherein students to impress the investigator may not have reported exact values for Internet use. Another limitation of this study was the tool (IAT) used to screen problematic Internet use, which did not survey the content of the Internet.

## 5. Conclusion

This study revealed high prevalence of problematic Internet use among Dilla University undergraduate students compared with the general population. In this study, being male, probable depression, and current use of khat or caffeinated drinks were significantly associated with problematic Internet use. The findings suggest that it is better to educate and focus on identifying high-risk groups to give special emphasis, providing psychological counseling, and, furthermore, students need to be educated about the safe, valuable, and healthy practices of Internet use. In addition, it is better to counsel on substance use and its consequences to overcome the anticipated increase in problematic Internet use.

## Figures and Tables

**Table 1:** Sociodemographic characteristics of Dilla University undergraduate students, Dilla, Ethiopia, in 2019.

Variables	Category	Frequency (761)	Percentage (90%)
Age	15–19	223	29.1
	20–24	503	66.1
	≥25	35	4.6

Sex	Male	455	59.8
	Female	306	40.2

Religion	Orthodox	500	65.7
	Muslim	127	16.7
	Protestant	124	16.3
	Others^*∗*^	10	1.3

Marital status	Single	661	86.9
	In relationship	60	7.9
	Married	20	2.6
	Others^*∗∗*^	10	1.3

Ethnicity	Oromo	261	34.3
	Amhara	355	46.6
	Tigre	35	4.6
	Wolaita	19	2.5
	Others^*∗∗∗*^	91	12.0

Living arrangement	With family	516	67.8
	Alone	172	22.6
	Others^*∗∗∗∗*^	73	9.6

Residence	Rural	542	71.2
	Urban	219	28.8

Academic year	First year	212	27.9
	Second year	193	25.3
	Third year	138	18.1
	Fourth year	114	15.1
	Fifth year	69	9.0
	Sixth year	35	4.6

Financial support	From family	672	88.3
	From relatives	65	8.5
	Others^*∗∗∗∗∗*^	24	3.2

^*∗*^Catholic, Wakifeta. ^*∗∗*^Separated, divorced, widowed. ^*∗∗∗*^Gamo, Kambat, Kore. ^*∗∗∗∗*^ With relatives, adopted. ^*∗∗∗∗∗*^From friends.

**Table 2:** Internet access and experience of Dilla University undergraduate students, Dilla, Ethiopia, in 2019.

Internet use experience (in months)	Never	10	1.3
	0 to 6	127	16.7
	6 to 12	171	22.5
	≥**12**	453	59.5

Internet use	≤5 hours	286	37.5
per day (in hours)	≥5 hours	475	62.4

Common mode of Internet access	Wi-Fi	222	29.1
	Broadband	101	13.2
	Mobile Internet	438	57.5

**Table 3:** Psychosocial and behavioral related characteristics of Dilla University undergraduate students, Dilla, Ethiopia, in 2019.

Depression	No	583	76.6
	Yes	178	23.4

Anxiety	No	548	72.0
	Yes	213	28.0

Social support	Poor	58	7.5
	Moderate	397	52.2
	Strong	307	40.3

Self-esteem	Low self-esteem	419	55.1
	High self-esteem	342	44.9

Peer influence	Lower peer pressure	421	56.4
	Higher peer pressure	340	43.6

**Table 4:** Factors associated with problematic Internet use among undergraduate students (bivariate and multivariate analysis) (*n* = 761), Dilla, Ethiopia, in 2019.

Variables	Categories	Problematic Internet use		Corollary (95% CI)	AOR (95% CI)
		Problematic	Normal		
Age	15–19	38	185	0.44 (0.20, 0.99)	0.53 (0.22, 1.25)
	20–24	99	404	0.53 (0.25, 1.12)	0.66 (0.29, 1.48)
	≥25	11	24	1.00	1.00

Sex	Male	105	315	1.83 (1.24, 2.70)	1.69 (1.12, 2.56)^*∗*^
	Female	43	263	1.00	1.00

Depression	No	77	506	1.00	1.00
	Yes	71	107	4.36 (2.97, 6.40)	3.61 (2.40, 5.43)^*∗∗*^

Anxiety	No	97	451	1.00	1.00
	Yes	51	162	0.68 (0.46, 1.01)	0.86 (0.56, 1.31)

Social support	Poor	67	240	2.73 (1.04, 7.13)	1.96 (0.72, 5.35)
	Moderate	76	321	2.32 (0.89, 6.02)	1.83 (0.68, 4.94)
	Strong	5	49	1.00	1.00

Self-esteem	Low self-esteem High self-esteem	82	337	0.98 (0.68, 1.41)	1.23 (0.83, 1.82)
		276	66	1.00	1.00

Current use of khat or caffeinated drinks	No	98	505	1.00	1.00
	Yes	50	108	2.38 (1.60, 3.55)	1.86 (1.21, 2.87)^*∗*^

1.00 reference. ^*∗*^*P* value less than 0.05. ^*∗∗*^*P* value less than 0.015. *P* value of Hosmer and Lemeshow test = 0.37, chi-square = 8.59, and df = 8.

## Data Availability

The data used to support the finding of this study are available from the corresponding author upon request.

## Abbreviations

CI: Confidence interval
CMD: Common mental disorders
E (DHS): Ethiopia (demographic health survey)
ETB: Ethiopian birr
EpiData: Epidemiological data
HADS: Hospital-based anxiety and depression scale
IAT: Internet Addiction Test
LAMIC: Low- and middle-income countries
MOSH: Ministry of Science and Higher education
SD: Standard deviation
SRS: Simple random sampling
SPSS: Statistical Package for the Social Sciences
SSA: Sub-Saharan Africa
UNICEF: United Nations Children's Fund
WHO: World Health Organization.
